# Increased lipocalin-2 expression in pulmonary inflammation and fibrosis

**DOI:** 10.3389/fmed.2023.1195501

**Published:** 2023-09-07

**Authors:** Apostolos Galaris, Dionysios Fanidis, Eliza Tsitoura, Paraskevi Kanellopoulou, Ilianna Barbayianni, Konstantinos Ntatsoulis, Katerina Touloumi, Sofia Gramenoudi, Theodoros Karampitsakos, Argyrios Tzouvelekis, Katerina Antoniou, Vassilis Aidinis

**Affiliations:** ^1^Institute for Fundamental Biomedical Research, Biomedical Sciences Research Center Alexander Fleming, Athens, Greece; ^2^Department of Respiratory Medicine, School of Medicine, University of Crete, Heraklion, Greece; ^3^Department of Respiratory Medicine, School of Medicine, University of Patras, Patras, Greece

**Keywords:** idiopathic pulmonary fibrosis (IPF), bleomycin (BLM), acute lung injury, transcriptomics, lipocalin-2 (LCN2)

## Abstract

**Introduction:**

Idiopathic Pulmonary Fibrosis (IPF) is a chronic, progressive interstitial lung disease with dismal prognosis. The underlying pathogenic mechanisms are poorly understood, resulting in a lack of effective treatments. However, recurrent epithelial damage is considered critical for disease initiation and perpetuation, via the secretion of soluble factors that amplify inflammation and lead to fibroblast activation and exuberant deposition of ECM components. Lipocalin-2 (LCN2) is a neutrophil gelatinase-associated lipocalin (NGAL) that has been suggested as a biomarker of kidney damage. LCN2 has been reported to modulate innate immunity, including the recruitment of neutrophils, and to protect against bacterial infections by sequestering iron.

**Methods:**

*In silico* analysis of publicly available transcriptomic datasets; ELISAs on human IPF patients' bronchoalveolar lavage fluids (BALFs); bleomycin (BLM)-induced pulmonary inflammation and fibrosis and LPS-induced acute lung injury (ALI) in mice: pulmonary function tests, histology, Q-RT-PCR, western blot, and FACS analysis.

**Results and discussion:**

Increased *LCN2* mRNA expression was detected in the lung tissue of IPF patients negatively correlating with respiratory functions, as also shown for BALF LCN2 protein levels in a cohort of IPF patients. Increased *Lcn2* expression was also detected upon BLM-induced pulmonary inflammation and fibrosis, especially at the acute phase correlating with neutrophilic infiltration, as well as upon LPS-induced ALI, an animal model characterized by neutrophilic infiltration. Surprisingly, and non withstanding the limitations of the study and the observed trends, *Lcn*2^−/−^ mice were found to still develop BLM- or LPS-induced pulmonary inflammation and fibrosis, thus questioning a major pathogenic role for *Lcn2* in mice. However, LCN2 qualifies as a surrogate biomarker of pulmonary inflammation and a possible indicator of compromised pulmonary functions, urging for larger studies.

## Introduction

Idiopathic pulmonary fibrosis (IPF) is a chronic, progressive interstitial lung disease characterized by the exuberant deposition of collagens and other ECM components by lung fibroblasts, leading to the distortion of lung architecture and the impairment of respiratory functions. The underlying mechanisms of the disease are poorly understood, resulting in a lack of effective treatments. However, epithelial damage is considered a key event initiating the pathogenesis of IPF, where repeated injury and/or abnormal repair of the epithelium trigger a cascade of signaling events that result in the recruitment and activation of immune cells, as well as the activation and accumulation of lung fibroblasts (1, 2).

Expression profiling of human IPF samples has been instrumental in the discovery of novel pathogenic genes and cellular pathways (3), that some were validated in animal models and some were translated into the clinic (4). In this context, we have recently developed Fibromine, a database and data mining tool, hosting all publicly available IPF transcriptomic (and proteomic) datasets (5), thus allowing the further exploitation of legacy data. Comparative analysis selected several hundred genes as differentially expressed in IPF, while an explainable machine learning phenotype classification algorithm prioritized 76 genes that include previously identified IPF expression hallmarks (e.g., Col1a1), IPF biomarkers (e.g., MMP7), as well as many genes previously shown to be involved in the pathophysiology of IPF (e.g., SPP1) (6). Among the novel, commonly identified deregulated genes in IPF was Lipocalin-2 (LCN2), also known as neutrophil gelatinase B-associated lipocalin (NGAL), as it was initially identified in neutrophilic granules in association with matrix metalloproteinase 9 (MMP9; gelatinase B) (7, 8). However, LCN2 secretion from other immune cells, as well as epithelial cells, has been reported (9, 10). LCN2 is considered an acute-phase protein, and increased LCN2 expression has been reported in different pathophysiological situations, including heart failure, kidney disease, and gut inflammation (10).

In the lung, increased LCN2 expression has been reported in subclinical pulmonary emphysema (11), chronic obstructive pulmonary disease (COPD) (12, 13), acute respiratory distress syndrome (ARDS) (14), as well as in patients with influenza A and SARS-CoV-2 virus infections (15). Not surprisingly, given their commonalities (16), higher LCN2 expression in bronchial epithelial cells of IPF patients has been also reported (17). Moreover, and more intriguingly, LCN2 has been suggested to mediate innate immune responses to bacterial infection by sequestrating iron (18), whereas both iron homeostasis (19), as well as microbiome regulation (20), have been linked with IPF pathogenesis. Therefore, in this report, we investigated a possible role for LCN2 in pulmonary inflammation and fibrosis, by using *in silico* analysis of publicly available transcriptomic datasets, examination of LCN2 protein levels in IPF patients, as well as *in vivo* mouse models of pulmonary inflammation and fibrosis.

## Materials and methods

### Datasets

All analyzed bulk-sequencing datasets (Supplementary Table S1) were sourced from Fibromine (5). scRNA sequencing (scRNAseq) datasets used in the study are detailed in Supplementary Table S2.

### Human patients

All studies were performed in accordance with the Declaration of Helsinki principles at the Department of Thoracic Medicine, University Hospital of Heraklion, and the demographics and clinical characteristics of the IPF patients can be found in Table 1. The diagnosis of IPF was based on ATS/ERS criteria or multidisciplinary discussion according to the Fleischer criteria (2, 21). Patients were anti-fibrotic naïve. All patients were evaluated with complete pulmonary function tests (PFTs) within 1 month of bronchoscopy. Lung volumes were measured using body plethysmography and the diffusion capacity (DLco, corrected for hemoglobin) using the single breath technique, and a computerized system (Jaeger 2.12; MasterLab, Würzburg, Germany). Patients were classified as non-smokers, current smokers, or former smokers (defined as having smoked a minimum of one cigarette a day for a minimum of 1 year, and stopping at least 6 months before presentation). All patients provided written informed consent. The study was approved by the Ethics Committees of the University Hospital of Heraklion (IRB numbers: 1045 and 17030).

**Table 1:** Demographics and clinical characteristics of IPF patients.

**Characteristic**	**IPF (*n = 26*)**
Age (yr) (Mean ± SD)	72.8 ± 7.3
**Gender**, ***n*** **(%)**	
Male	25 (96.1%)
Female	1 (3.9%)
**Pulmonary function tests (mean** ±**SD)**	
DLCO%	56.2 ± 19.4
FEV1/FVC%	85.4 ± 4.7
KCO	94.1 ± 21.5
**Hematological analysis (%) (mean** ±**SD)**	
Macrophages	83.1 ± 9.8
Lymphocytes	7.6 ± 7.6^*^
Polymorphonuclear	7.5 ± 6.4
Eosinophils	1.3 ± 1.61
BALF LCN2 (ng/mL) eosinophils	58.9 ± 52.3

^*^FVC%, Forced vital capacity percent predicted; DLCO%, Carbon monoxide diffusing capacity percent predicted; FEV1%, Forced expiratory volume in 1-s percent predicted.

### Mice

All mice were bred at the animal facilities of the Alexander Fleming Biomedical Sciences Research Center under specific pathogen-free conditions. Mice were housed at 20–22°C, 55 ± 5% humidity, and a 12-h light–dark cycle; water and food were given *ad libitum*. Mice were bred and maintained in their respective genetic backgrounds for more than 10 generations. All experiments performed on mice for this project were in line with the ARRIVE guidelines and were approved by the Institutional Animal Ethical Committee (IAEC) of the Biomedical Sciences Research Center “Alexander Fleming” (#373/375), as well as the Veterinary service and Fishery Department of the local governmental prefecture (#5508). Lipocalin-2 deficient mice (*Lcn*2^−/−^) were procured from the Jackson Laboratory (#024630) and were maintained in a C57Bl6 genetic background for over 10 generations; genotyping was performed as previously published (18). Mice were humanely euthanized in a gradually filled CO_2_ chamber.

### BLM-induced pulmonary fibrosis

Pulmonary fibrosis was induced through the administration of bleomycin (BLM, 0.8 U/Kg of body weight; Nippon Kayaku) to anesthetized mice (intraperitoneal; ketamine/xylazine/atropine, 100/10/0.05 mg/Kg, respectively) via the oropharyngeal (OA) track, as previously described (22). In brief, mice were carefully placed on a plastic wall upon anesthesia. Their tongue was pulled out with forceps to get a better view of the trachea. The nares were blocked to force inhalation, and the bleomycin, diluted in normal saline (~50 μl for each mouse), was directly delivered to the oropharyngeal cavity using a conventional pipette tip. Normal saline was administered in the same way to littermate mice used as controls 3, 7, and 14 days after bleomycin (or saline) administration, at the peak of BLM-induced disease (which spontaneously resolves at d21).

### Lipopolysaccharide (LPS)-induced acute lung injury (ALI)

The acute lung injury (ALI) model was performed using LPS delivered by inhalation, as previously described (23). In brief, bacterial lipopolysaccharides (LPS) from *Pseudomonas aeruginosa* (serotype 10, Sigma, St. Louis, MO, USA) were dissolved in normal saline at a concentration of 2 mg/ml. A total of 5 ml of this solution was administered into a chamber containing 5–7 mice via a custom-made nebulizer at an oxygen flow rate of 4 lt/min for 25 min. Normal saline was administered to the control mice. All measures were taken to minimize animal suffering; however, during the protocol, no anesthetics were used as no invasive or painful techniques were performed. After the induction of ALI, the condition of the animals was checked every 2 h during the light period. Mice were euthanized 24 h after the induction of ALI.

### Respiratory functions

The respiratory functions were examined with FlexiVent (Scireq), following the manufacturer's instructions and as previously published (22).

### Analyses of samples

Blood was collected through the portal vein and placed into tubes containing 0.5 M EDTA at a concentration of 10%^v^/_v_. Then, it was centrifuged for 20 min at 2.000 *g* at 4°C, and the plasma was transferred in new siliconized tubes. BALF was obtained by lavaging the airways with 3 ml of normal saline using a cannula through the trachea (three times; 1 mL each). Then, BALF was centrifuged for 15 min at 1.200 *g* at 4°C. The first 1 ml of the BALF was transferred without the cells into a new siliconized tube. The other 2 ml were discarded; the cells were pooled and treated with GEYS solution for 10 min in ice. Then, they were centrifuged for 10 min at 1.200 *g* at 4°C, the suspension was discarded, the cell pellet was re-suspended in fresh PBS, and the cells were counted under an inverted microscope using a Neubauer chamber. The left lung lobe was cut and instantly transferred into liquid nitrogen for RNA and protein extraction. The remaining lobes were filled with formalin (143091.1214, AppliChem), to be later mounted into paraffin. Additionally, total protein concentration was estimated in the BALF using Bradford reagent (Cat.no.: 39222.03, SERVA) following the manufacturer's instructions.

### Flow cytometry

Mice were euthanized under deep anesthesia followed by exsanguination. Then, BALF was collected via tracheotomy by injecting and slowly withdrawing 3 ml (3 times; 1 mL each) of phosphate-buffered saline (PBS). The cells were collected via 10 min centrifugation at 1,200 rpm at 4°C, and they were treated with 1 mL of Gey's Solution for 2–3 min. The Gey's Solution was removed after a 10 min centrifugation at 1,200 rpm at 4°C, and the cells were resuspended in 1XPBS/1 %FBS and counted manually under a reversed light microscope using an improved Neubauer hemacytometer according to common procedures. Next, the cells were centrifuged at 1,200 rpm for 10 min at 4°C. The cell pellets were resuspended in 50 μl blocking buffer (1XPBS with 1% FBS and 1:400 CD16/32) for 10 min. Then, 100 μL of PBS was added to each sample, and the cells were collected via 5 min centrifugation at 1200 rpm at 4°C. The cells were resuspended and stained in the desired concentrations of antibodies in 1XPBS + 1%FBS for 30 min. Then, 100 μL of PBS was added to each sample, and the cells were collected via 5 min centrifugation at 1200 rpm at 4°C. Finally, the cells were resuspended in 250 μl filtered PBS, and data were acquired on a BD FACSCanto TM II flow cytometer using BD FACSDiva software (BD Biosciences). The analyses of the RAW data were performed with the FlowJo software (TreeStar, Ashland, OR).

### RNA extraction and real-time PCR

The upper half of the left lobe isolated from the animals was homogenized in 1 mL of Trizol (TR118, Molecular Research Center) followed by total RNA extraction according to the manufacturer's instructions. A total of 2 μg of total RNA were used for cDNA construction using M-MLV reverse transcriptase (28025-013, Invitrogen) according to the manufacturer's instructions. Real-time polymerase chain reaction (RT-PCR) was performed using SoFAst EvaGreen Supermix on a Bio-Rad CFX96 Touch™ real-time PCR detection system (Bio-Rad Laboratories Ltd, CA, USA). Values were normalized to β2-microglobulin (B2M) and the primers used are: Lcn2 (F: 5′-GGG AAA TAT GCA CAG GTA TCC TC-3′; R: 5′- CAT GGC GAA CTG GTT GTA GTC-3′) and B2M (F: 5′-TTC TGG TGC TTG TCT CAC TGA-3′; R: 5′-CAG TAT GTT CGG CTT CCC ATT C-3′).

### Protein extraction and Western blot analysis

The lower half of the left lobe was homogenized in 100 μL of homemade RIPA cell lysis buffer (20 mM Tris-HCl pH = 7.5, 150 mM 5M NaCl, 2 mM 0.5 M EDTA, 1 mM 0.5 M EGTA, 0.5% Sodium Deoxycholate, 0.1% SDS, 1% N-P40) containing protease inhibitor mixture (Cat. No: 11836170001, Roche) using a manual tissue grinder, and lysates were spun n at 10.000 rpm for 10 min at 4°C. Protein concentration was estimated by Bradford reagent (Cat. no.: 39222.03, SERVA), and 10 μg of total protein was prepared for immunoblotting in the final volume of 15 μl. In detail, protein mixtures were incubated at 100°C for 5 min, and they were immediately spun and electrophoresed in SDS-PAGE gel. Proteins were then transferred onto nitrocellulose blotting membrane (GE10600002, Amersham, Germany), and the membranes were incubated in 1% BSA-1‰ Tween20 PBS in 1:1200 rabbit anti-mouse lipocalin-2 antibody (ab63929, Abcam) and 1:1,200 goat anti-mouse actin antibody (sc-1615, Santa Cruz Biotechnology) O/N at 4°C. The next day, the membranes were washed in 1‰ Tween20 PBS followed by incubation with 1:20.000 secondary antibodies (anti-rabbit: 925-68073, LI-COR; anti-goat: 925-32214, LI-COR) in 1% BSA-1‰ Tween20 PBS. The blot was visualized in an Odyssey DLx Imaging System (LI-COR).

### Immunohistochemistry

Fixed lung tissues were mounted into paraffin; 4 μm slices were cut and placed on slides. Then, hematoxylin/eosin (H&E) staining was performed as previously described (22). In brief, the slices were deparaffinized at 60°C for 2 h followed by xylene washes and hydrated in gradual ethanol concentrations. The slices were stained against Lcn2 (ab63929, Abcam) in 1:200 concentration. Peroxidase conjugated secondary antibody (4010-05, Southern Biotech) and DAB kit (SK-4100, Vector Laboratories, Inc.) were used to visualize Lcn2 in the lung tissue slices.

### ELISA

LCN2 levels were estimated in human and murine BALF using a commercially available ELISA kit (EA100541, OriGene Technologies Inc.), according to the manufacturer's instructions.

### *In silico* analyses

Differential gene expression analysis results produced during Fibromine creation (5) were used for volcano plot creation. Respective boxplots summarize *LCN2* expression in terms of log2 fold change, while depicted datasets had a statistically significant difference between the compared groups (IPF_vs_Ctrl; Bleomycin_vs_Ctrl). Absolute fold change (FC) of at least 1.2 and FDR-corrected *p* < 0.05 were selected as thresholds for differential expression. The correlation of *LCN2* expression values with those of spirometry measurements was examined using Spearman's correlation test. An absolute rho value of at least 0.5 was considered the threshold of a strong relationship, while a *p* < 0.05 was required for a relationship to be deemed significant. Visualizations were performed using packages ggplot2 (v.3.3.5) and ggrepel (v.0.9.1).

Single-cell RNA-seq data were found at GSE136831 (24), (GSE135893_ILD_annotated_fullsize.rds.gz) (25), as well as in the GitHub repositories (26, 27). All downstream described processes were completed with the R package Seurat (4.0.5) (28, 29).

For GSE136831, already filtered data were log normalized using a scaling factor of ten thousand (*NormalizeData*), and highly variable features (HVG) were retrieved (*FindVariableFeatures*) and scaled (*ScaleData*). Linear dimensionality reduction (PCA) (*RunPCA*) was followed by the creation of the closest neighborhood graph (*FindNeighbors*) using the first 7 principal components, as proposed by the median of all findPC methods output (30). Clusters were identified using Louvain clustering with a resolution of 1.3 (*FindClusters*). Cell typing information provided along with the count data was adopted. Non-linear dimensionality reduction was performed using Uniform Manifold Approximation and Projection (UMAP) (*RunUMAP*). As its name implies, UMAP is a non-linear method for reducing the dimensions of a dataset based on manifold calculation (31). Although not developed for scRNA-seq data *per se*, it is a method of choice for the analysis of such data yielding reproducible results in fast running times (32). Taking into consideration the same number of principal components as above results in a visualization very similar to that of the initial publication. Batch correction of any kind was not performed as proven unnecessary during the original data analysis.

From the GSE135893 object, IPF and control originating cells were maintained, while read counts were log normalized with a scaling factor of ten thousands before any downstream analysis (*NormalizeData* function). Mayr et al. lung dataset object was analyzed for IPF and control cells only, while barcodes that were assigned an “empty” cell type were removed. Log normalization with a 10,000 scaling was applied (*NormalizeData* function). Similarly, barcodes assigned a “NA” or “Low-Quality Cells” cell type were removed from the Strunz et al. whole lung dataset object before downstream analysis.

For all single-cell data differential expression analyses, the Wilcoxon rank-sum test was applied (*FindMarkers*), while an absolute FC of at least 1.2 and a Bonferroni-corrected *p* < 0.05 were set as significant thresholds.

### Statistics

Statistical analysis was performed using the GraphPad Prism software (v8.0, GraphPad, San Diego, California, USA), as explicitly indicated in each figure legend.

## Results

### Increased *LCN2* expression in IPF patients negatively correlates with respiratory functions

**Figure 1:**
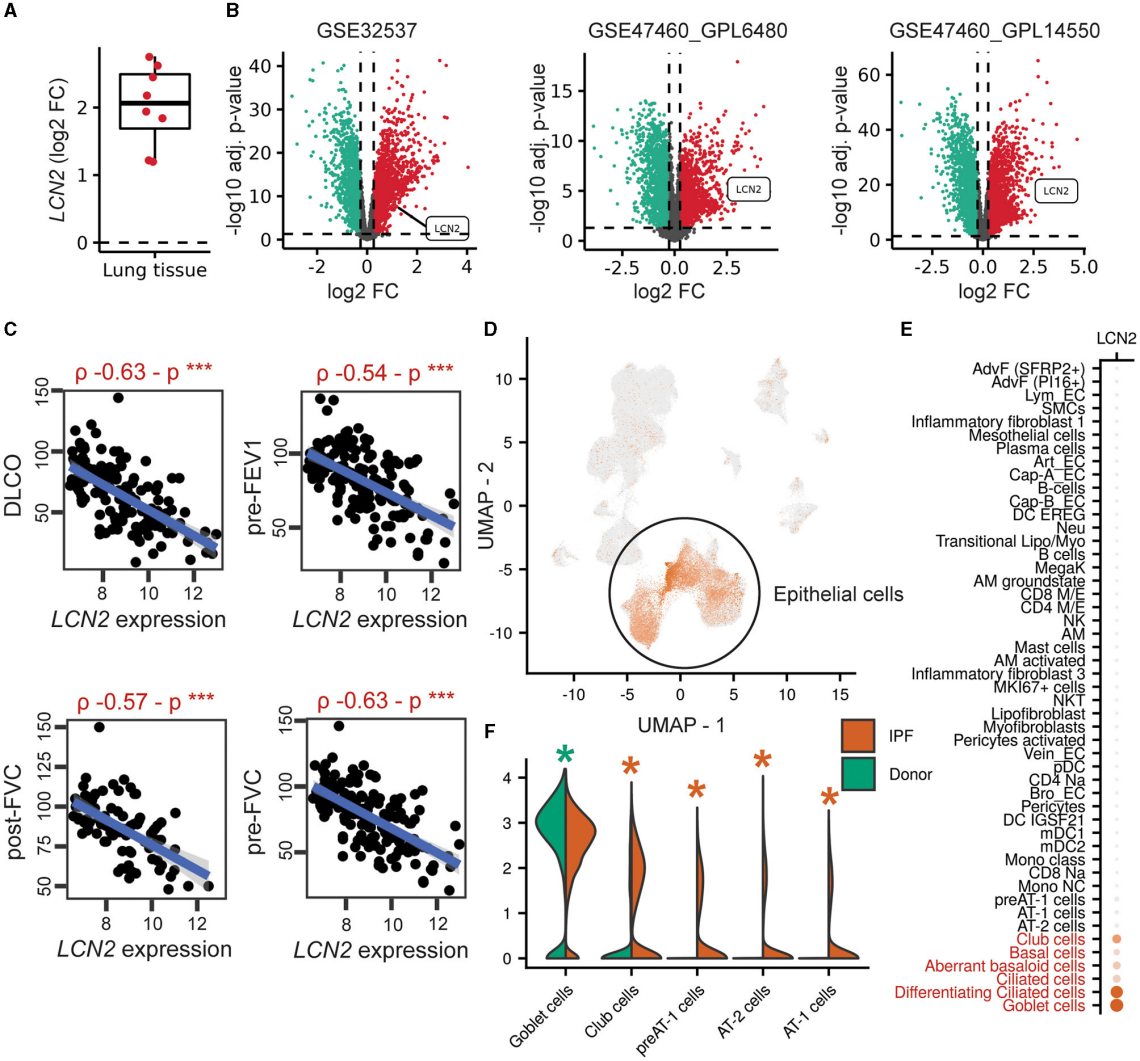
Increased *LCN2* mRNA expression in lung epithelial cells of IPF patients, negatively correlating with their respiratory functions. **(A)** Mean differential *LCN2* mRNA expression in IPF patients' cohorts/datasets, detailed in Supplementary Table S1. **(B)** Volcano plots of differentially expressed genes (FC > 1.2; FDR adjusted *p* < 0.05) in the three largest transcriptomics datasets of **(A)** interrogating the expression of 115/44, 28/15, 84/75 IPF patients and controls, respectively (Supplementary Table S1). **(C)** Spearman's correlation plots of *LCN2* expression with spirometry measurements from the GSE47460_GPL14550 cohort (***p < 0.01). **(D)** Dimensionality reduction plot localizing *LCN2* expression in pulmonary epithelial cells originating from 4 IPF patients and 6 controls (26). **(E)** Dot plot of the same dataset depicting cell type-specific *LCN2* expression. The Wilcoxon rank-sum test comparing each cell type with the rest validated *LCN2* as a marker gene of the cell types marked in red font (FC > 1.2; Bonferroni adjusted *p* < 0.05). **(F)** Per cell type differential expression analysis between cells of different phenotype (IPF vs. control origin) using the Wilcoxon rank-sum test (*FC ≥ 1.2; Bonferroni-corrected *p* < 0.05; *upregulated in IPF; *downregulated in IPF).

To explore a possible involvement of *LCN2* in IPF, *LCN2* expression was interrogated in IPF transcriptomic datasets (Supplementary Table S1), sourced from Fibromine (www.fibromine.com), a database and data mining tool for target discovery in IPF (5). Using absolute fold change of at least 1.2 and FDR-corrected *p* < 0.05, widely accepted thresholds for the selection of differentially expressed genes, the expression of *LCN2* was found to be significantly increased in most datasets interrogating gene expression in the lungs of IPF patients in comparison with control individuals (Supplementary Table S1; Figure 1A). Indicatively, *LNC2* presented with a natural scale fold change of 2.3, 4.5, and 3.9 in three of the largest ones (Figure 1B; Supplementary Table S1). Importantly, *LCN2* expression negatively correlated with the respiratory functions (DLCO, FVC, and FEV1) of IPF patients in the same datasets (Figure 1C; Supplementary Figures S1A, B).

To examine the cell specificity of *LCN2* expression in fibrotic lungs, we re-analyzed data from three publicly available single-cell RNA seq (scRNAseq) datasets of human origin (Supplementary Table S2) (24–26). *LCN2* was found in all three data collections (Figure 1D; Supplementary Figures S1C, D), primarily expressed in epithelial cells, including goblet, ciliated, basal, club, and aberrant basaloid cells (Figures 1D, E; Supplementary Figures S1E, F). Comparing cell types between phenotypes (IPF and control), *LCN2* was found over-expressed mostly in alveolar type 1 and 2 cells (AT1 and AT2) (Figure 1F; Supplementary Figures S1G, H). However, *LCN2* expression from neutrophils, as shown in other pathological contexts summarized by the CellMarker2.0 database (33) (Supplementary Table S3), cannot be excluded, given the low representation of neutrophils in human IPF scRNAseq datasets.

**Figure 2:**
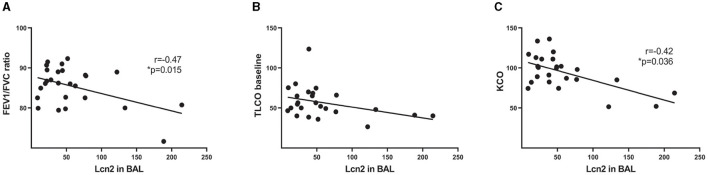
LCN2 BALF levels of IPF patients negatively correlate with their respiratory functions. **(A–C)** Spearman's correlation plots of LCN2 levels, as measured using a commercially available ELISA, with the ratio of forced expiratory volume (FEV)/forced vital capacity (FVC) **(A)**, with a transfer capacity of the lung for the uptake of carbon monoxide (TLCO) **(B)** and with carbon monoxide transfer coefficient (KCO) **(C)**. Statistical significance was assessed with Spearman's r = −0.47, −0.42 as indicated; * denotes *p* < 0.05.

To validate the *in silico* findings, we estimated LCN2 levels in the bronchoalveolar lavage fluid of 26 IPF patients (Table 1), with a commercially available ELISA kit. As shown *in silico* for mRNA levels in the lung tissue of IPF patients (Figure 1), LCN2 BALF levels of IPF patients negatively correlated with their respiratory functions (FEV1/FVC, TLCO, and KCO) (Table 1; Figures 2B, C).

Therefore, IPF is associated with increased *LCN2* expression, predominantly in pulmonary epithelial cells, negatively correlating with impaired lung functions.

### Increased *Lcn2* expression upon pulmonary inflammation and fibrosis in mice

**Figure 3:**
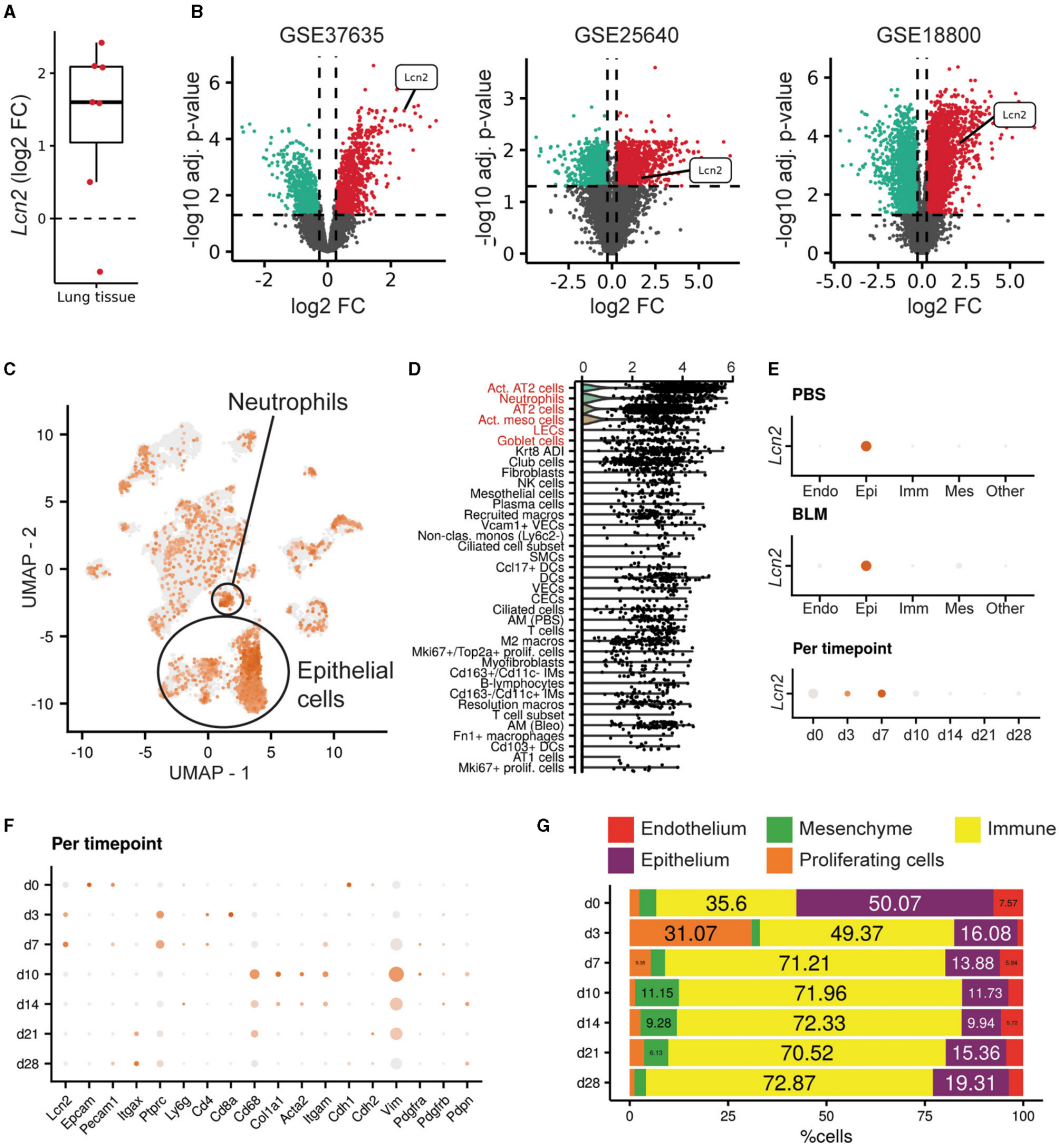
Increased *Lcn2* expression in mouse lungs post-BLM-induced pulmonary fibrosis. **(A)** Mean differential *Lcn2* mRNA expression in different transcriptomics BLM datasets (Supplementary Table S1). **(B)** Volcano plots of the three major datasets of **(A)**. **(C)** Feature plot showing *Lcn2* expression in the mouse lung (27). **(D)** Dot plot revealing the cell type expression pattern of *Lcn2* (in decreasing order of importance). The Wilcoxon rank-sum test comparing each cell type with the rest validated *Lcn2* as a marker gene of the cells types marked in red font (FC > 1.2; Bonferroni adjusted *p* < 0.05). **(E)** Separate examination of the control (PBS) and fibrotic (BLM) cells further supports the epithelial origin of *Lcn2*. **(F)** Per timepoint examination of cell population markers. **(G)** Bar plot depicting the changes in the mouse lung major cell populations, as defined by scRNAseq analysis clustering and cell typing, across timepoints of BLM administration.

To examine *Lcn2* expression in the lungs of mice post-bleomycin (BLM)-induced pulmonary inflammation and fibrosis, a widely used animal model of pulmonary fibrosis (4, 22, 34), we mined the relative transcriptomic datasets from Fibromine (Supplementary Table S1), as in IPF patients. *Lcn2* was found over-expressed in most datasets when comparing the fibrotic phase of the model to control samples (Supplementary Table S1; Figure 3A), with indicative natural scale fold change scores of 5.4, 3, and 4.2 (Figure 3B). Moreover, re-analysis of a publicly available murine scRNAseq dataset (Supplementary Table S2) (27) indicated that, as in the human lung, *Lcn2* is highly expressed mainly by epithelial cells, as well as neutrophils (Figures 3C, D). More specifically, classical and activated AT2 cells, neutrophils, goblet, and activated mesothelial cells, as well as lymphatic endothelial cells (LECs), were marked by *Lcn2* expression (Figures 3C, D). Similar results were revealed from the CellMarker2.0 database query (33), where *Lcn2* was defined as a marker of murine lung neutrophils and AT2 cells (Supplementary Table S3). Importantly, the highest *Lcn2* expression was detected during the earlier inflammatory phase of the model (Figures 3E–G), which is characterized by epithelial damage and neutrophilic inflammation.

**Figure 4:**
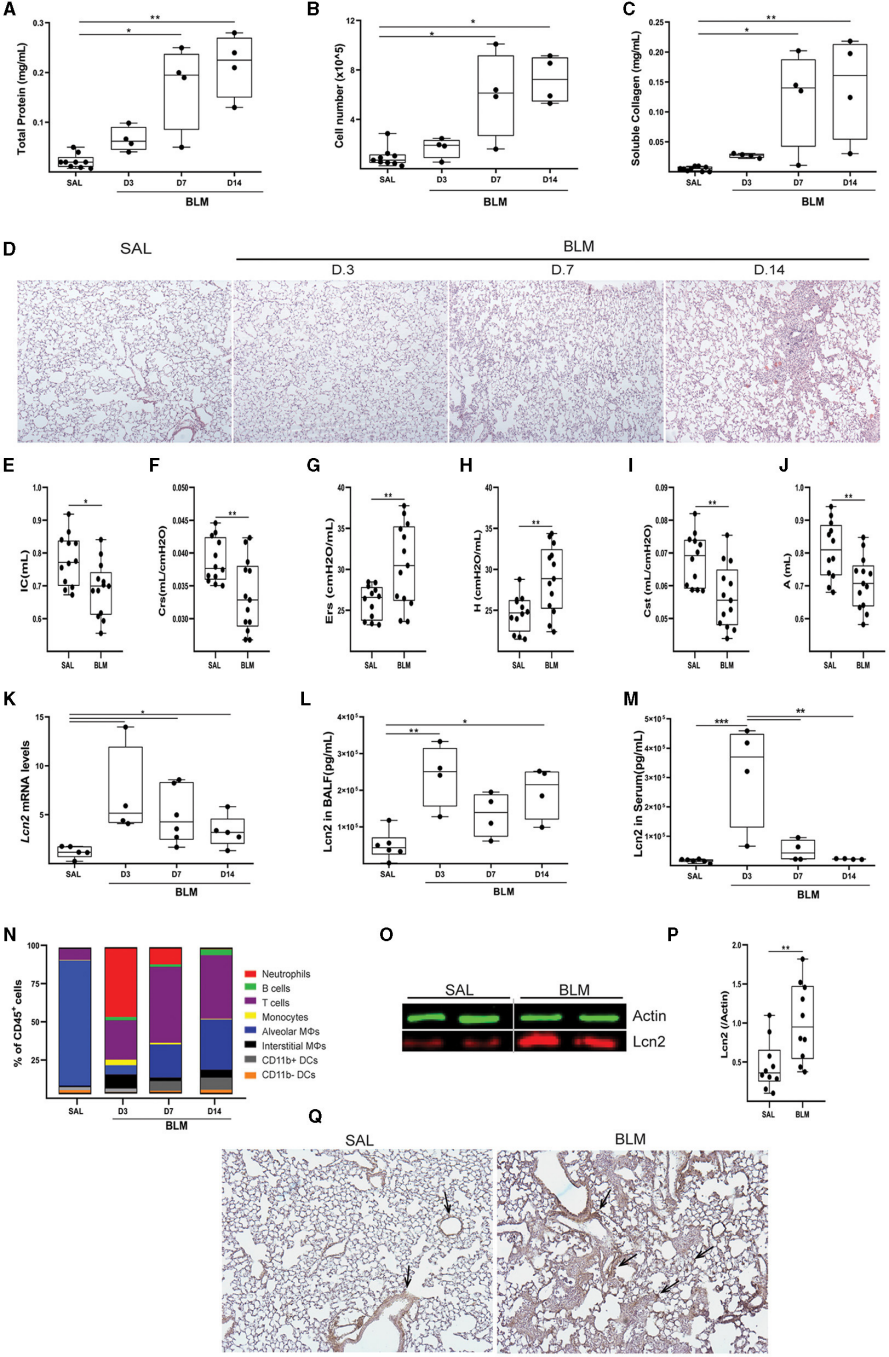
Increased *Lcn2* expression in mouse lungs during the development of BLM-induced pulmonary inflammation and fibrosis. **(A)** Total protein concentration in BALFs, as determined using the Bradford assay. **(B)** Inflammatory cell numbers in BALFs, as counted with a hemacytometer. **(C)** Soluble collagen levels in the BALFs as detected with the Direct Red assay. Statistical significance was assessed with one-way ANOVA; */** denote *p* < 0.05/0.01 respectively. **(D)** Representative images from H&E-stained lung sections of murine lungs at 3, 7, and 14 d post-BLM administration (×10). **(E–J)** Respiratory functions were measured with FlexiVent, 14 days post-BLM; mean respiratory system compliance (Crs); mean respiratory system elastance (Ers); mean tissue elastance (H); mean static lung compliance (Cst); mean total lung capacity (A). Cumulative results from three independent experiments; statistical significance was assessed using the Mann–Whitney test; */** denote *p* < 0.05/0.01, respectively. **(K)** *Lcn2* mRNA expression was interrogated using Q-RT-PCR; Values were normalized over the expression of the housekeeping gene *B2m* and presented as fold change over control. **(L, M)** Lcn2 concentration in BALF **(L)** and serum **(M)** of mice at 3, 7, and 14 d post-BLM administration. Lcn2 levels were measured using a commercially available ELISA kit; Statistical significance was assessed with one-way ANOVA, */**/*** denote *p* < 0.05/0.01/0.001 respectively. **(N)** Bar plot showing the percentage of immune cell populations in the murine lung post-BLM; the employed gating strategy is described in Supplementary Figure S2. **(O)** Representative Western blot of Lcn2 expression (red) in fibrotic lungs, 14 d post-BLM. **(P)** Densitometry analysis of Lcn2 expression, normalized to the expression of Actin (green); cumulative result from two independent experiments; statistical significance was assessed with unpaired *t*-test; ** denotes *p* < 0.01. **(Q)** Representative images of two independent experiments, from immunohistochemistry for Lcn2 in control (SAL) and fibrotic (BLM) murine lung tissue (×10).

To validate the *in silico* mouse results, we examined *Lcn2* expression during the development of BLM-induced pulmonary inflammation and fibrosis. To this end, BLM (0.8 U/Kg) was administered by oropharyngeal aspiration to 8–10-wk-old C57Bl6 mice, which were then euthanized 3, 7, and 14 days post-BLM administration, timepoints corresponding to the inflammatory (3, 7) and fibrotic (14) phases of the disease (which resolves at 21 d; not shown). As expected, BLM administration resulted in the vascular leak and pulmonary edema, as indicated by the total protein concentration of the bronchoalveolar lavage fluid (BALF), determined with the Bradford assay (Figure 4A), as well as in inflammation, as indicated by the inflammatory cells in the (BALF) (Figure 4B). Soluble collagen levels in the BALF, as determined with the Direct Red assay, were also found gradually increasing in fibrotic lungs (Figure 1C). The H&E staining performed in lung sections of murine lungs post-BLM administration revealed the increasing presence of peribronchiolar and parenchymal fibrotic regions (Figure 4D). Moreover, the development of pulmonary fibrosis was reflected in the impairment of respiratory functions, as quantified with FlexiVent (Figures 4E–J). The development of BLM-induced pulmonary fibrosis and the impairment of respiratory functions were associated with increased lung tissue *Lcn2* mRNA expression, as detected with Q-RT-PCR, in all phases of the disease, but especially in the acute inflammatory phase (Figure 4K). A similar profile was detected in the Lcn2 protein concentrations in the BALF (Figure 4L), while the increased concentration in the serum of the same mice was only detected in the acute phase. To possibly correlate Lcn2 levels with immune cell populations in the BALF post-BLM administration, a multicolor FACS analysis was performed, quantifying 10 distinct immune cell types; the employed gating strategy is described in detail in Supplementary Figure S2. FACS indicated an abundance of neutrophils in the acute inflammatory phase post-BLM (Figure 4O), at the peak of Lcn2 expression. However, increased Lcn2 protein levels could still be detected in the fibrotic lung tissue 14 d post-BLM (Figures 4P, Q), while Lcn2 immunostaining was localized in epithelial cells and fibrotic regions, constitutive expression was detected from the bronchial epithelium (Figure 4R).

**Figure 5:**
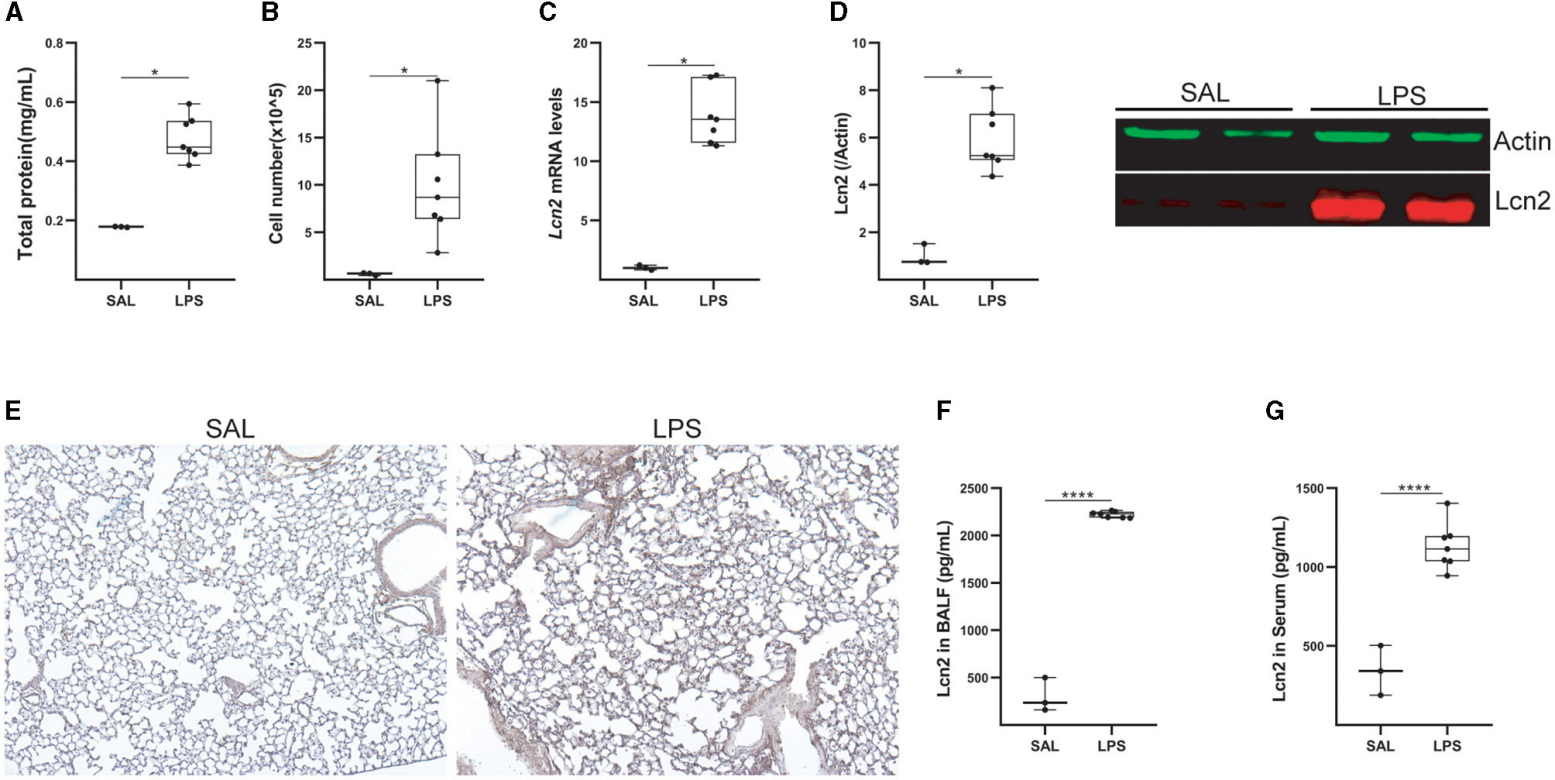
*Lcn2* expression is upregulated during LPS-induced Acute Lung Injury (ALI). **(A)** Total protein concentration in BALF, as determined using the Bradford assay. **(B)** Inflammatory cell numbers in BALF, from saline and LPS-treated mice, as counted with a hemacytometer. **(C)** *Lcn2* mRNA expression was interrogated with Q-RT-PCR; Values were normalized over the expression of the housekeeping gene *B2m* and presented as fold change over control; representative results from three independent experiments. **(D)** Western blot of Lcn2 expression (red) in lungs from mice with LPS-induced ALI, followed by densitometry analysis of Lcn2 expression, normalized to the expression of Actin (green). **(E)** Representative images from immunohistochemistry for Lcn2 in lungs from control (SAL) and LPS-treated mice (×10). **(F, G)** Lcn2 levels in BALF **(F)** and serum **(G)** of mice were estimated using ELISA; statistical significance was assessed using the Mann–Whitney test; */**** denote *p* < 0.05/0.0001.

To confirm Lcn2 as a marker of pulmonary inflammation, we then examined Lcn2 levels upon LPS-induced acute lung injury (ALI). LPS was administered (5 mL; 2 mg/mL) via a nebulizer (flowrate 4 lt/min) to WT C57Bl6 mice, that were euthanized 24 h later. The development of ALI, as indicated by the vascular leak (Figure 5A) and the infiltration of inflammatory cells (Figure 5B; mostly neutrophils; data not shown) (35), was associated with increased *Lcn2* mRNA (Figure 5C) and protein levels (Figures 5D, E) in the lung tissue. The increased Lcn2 expression upon ALI was also reflected in the BALF and sera of the same mice (Figures 5F, G).

Therefore, Lcn2 is a marker of pulmonary inflammation in mice, correlating with epithelial damage and neutrophilic infiltration.

### Genetic dissection of the role of Lcn2 in pulmonary inflammation and fibrosis in mice

**Figure 6:**
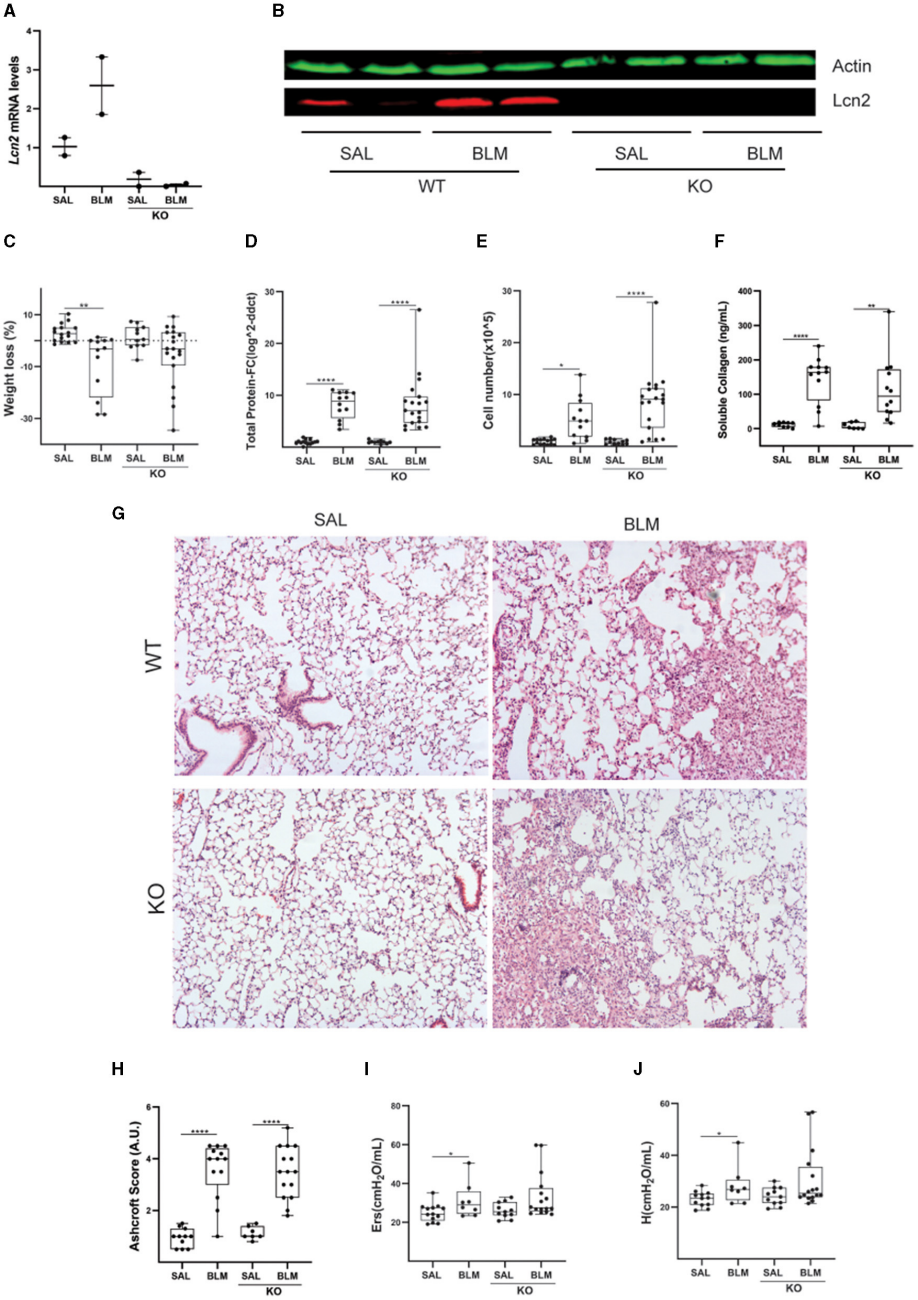
*Lcn2* genetic deficiency has minor effects in bleomycin (BLM)-induced pulmonary inflammation and fibrosis. **(A)** *Lcn2* mRNA expression was interrogated with Q-RT-PCR; Values were normalized over the expression of the housekeeping gene *B2m* and presented as fold change over control; **(B)** Representative Western blot of Lcn2 expression (red) in lungs from WT and KO mice treated with BLM confirming the global depletion of *Lcn2* in KO mice. **(C)** Weight loss post-BLM administration. **(D)** Total protein concentration in BALFs, as determined with the Bradford assay. **(E)** Inflammatory cell numbers in BALFs, as counted with a hemacytometer. **(F)** Soluble collagen levels in the BALFs as detected with the Direct Red assay. **(G)** Representative H&E-stained lung sections (×10). **(H)** Ashcroft scoring of disease severity. **(I, J)** Indicated respiratory functions were measured with FlexiVent; statistical significance was assessed with one-way ANOVA; */**/**** denotes *p* < 0.05/0.01/0.0001.

To dissect a possible role for Lcn2 in pulmonary inflammation and fibrosis, we then investigated the effects of BLM-induced pulmonary inflammation and fibrosis on *Lcn2* ubiquitous knockout mice (KO); the lack of Lcn2 expression in KO mice was verified using Q-RT-PCR and Western blot analysis (Figures 6A, B). The BLM-induced weight loss, an indicator of overall systemic health, did not reach statistical significance in *Lcn2*^−/−^ mice (Figure 6C); however, no statistically significant changes were detected in vascular leak (Figure 6D), inflammation (Figure 6E), or soluble BALF collagen (Figure 6F). Accordingly, no major statistically significant differences were detected in the associated distortion of lung architecture (Figures 6G, H). However, BLM-induced impairment of respiratory functions did not reach statistical significance in *Lcn2*^−/−^ mice (Figures 6I, J), suggesting again, as the human data, a possible negative correlation of Lcn2 expression with respiratory functions.

Given the suggested role of Lcn2 in metabolic disorders and obesity (36) and the correlation between IPF and obesity in patients (37), the effect of obesity-driven microbiome changes in the lungs (38), as well as the suggested role of Lcn2 in iron sequestration and microbiome regulation, we next investigated the role of Lcn2 in the pathogenesis of pulmonary fibrosis in obese mice, following the high-fat diet (HFD) feeding for 13 weeks, in comparison with mice fed a matched control diet. No statistically significant changes in disease severity were observed either, although a clear trend of disease attenuation was observed (Supplementary Figures S3A–D), as opposed to lean mice.

**Figure 7:**
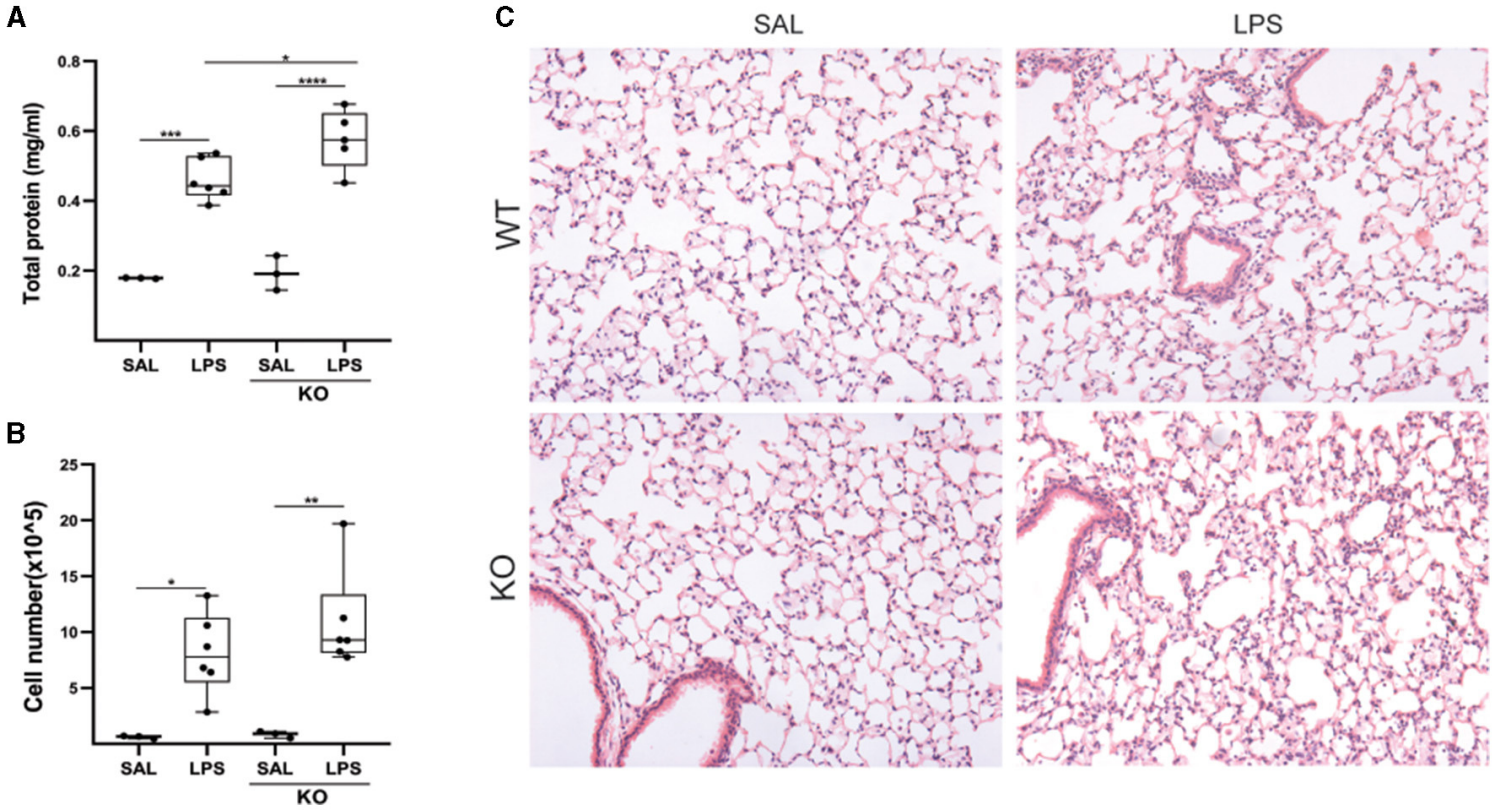
*Lcn2* genetic deficiency has minor effects on LPS-induced pulmonary inflammation. **(A)** Total protein concentration in BALFs, as determined with the Bradford assay. **(B)** Inflammatory cell numbers in BALFs, as counted with a hemacytometer; **(C)** Representative H&E-stained sections of murine lungs of WT and Lcn2 KO mice (×10); statistical significance was assessed with one-way ANOVA; */**/***/**** denotes *p* < 0.05/0.01/0.001/0.0001.

Moreover, given the increased *Lcn2* expression in the acute phase post-BLM administration (Figure 4), as well as following LPS-induced ALI (Figure 5), we then examined a possible role of Lcn2 in acute inflammation by administering LPS in *Lcn2*^−/−^ and control wt mice. *Lcn2*^−/−^ mice presented with increased pulmonary edema (Figure 7A), but no significant changes in inflammation (Figures 7B, C).

Therefore, despite observed trends in *Lcn2*^−/−^ mice, no solid conclusions on the role of Lcn2 in pulmonary inflammation and fibrosis could be drawn upon disease modeling in mice, in these settings.

## Discussion

In this report, increased *LCN2* mRNA expression has been detected *in silico* in most available transcriptomics datasets at Fibromine.com (Figure 1; Supplementary Table S1). The *in silico* approach, given the availability of datasets in Fibromine, emerges as a valuable surrogate tool for the identification of the expression levels of genes under investigation. Moreover, given the multiple available datasets/human samples, the method is more practical and valuable than the usual practice, i.e., individual RT-PCRs in a limited number of IPF samples.

*LCN2* mRNA expression levels in IPF patients negatively correlated with respiratory functions (Figure 1); accordingly, LCN2 BALF levels negatively correlated with patients' respiratory functions (FEV1/FVC, TLCO, and KCO) of a cohort (n=26) of IPF patients (Figure 2), in agreement with a previous study (17). However, much larger clinical studies will be needed to possibly associate LCN2 expression levels, in both sera and BALF, with respiratory functions and other specific pathophysiological disease attributes. A meta-analysis of publicly available scRNAseq datasets indicated the lung epithelium as the major source of LCN2 in the fibrotic lung (Figure 1), as previously shown with immunocytochemistry (17). Recurrent epithelial damage is considered the initiating insult of IPF pathogenesis, and acute exacerbation of IPF is characterized by increased alveolar epithelial cell injury, suggesting that future studies on LCN2 and IPF should include the evaluation of LCN2 levels in patients with acute IPF exacerbation and the correlation with other epithelial injury markers.

Similar results were obtained in BLM-induced pulmonary fibrosis in mice (Figures 3, 4), further indicating higher *Lcn2* expression in the acute phase of the disease, following BLM-induced epithelial damage and correlating with neutrophilic inflammation. Lcn2 levels declined at the fibrotic phase, although remained higher than controls, as is the case for various inflammatory markers, e.g., TNF (39). Moreover, and in agreement with an acute role for Lcn2, neutrophilic infiltration upon LPS-induced ALI was also correlated with higher Lcn2 expression (Figure 5).

However, despite the increased Lcn2 expression upon BLM- or LPS-induced lung damage, no statistically significant changes were observed upon BLM or LPS administration to *Lcn2*^−/−^ mice (Figures 6, 7; Supplementary Figure S3), suggesting that either Lcn2 does not have a major pathogenic role or Lcn-2 can have different roles in different cell populations, masked in the ubiquitous knockout mice, and that a cell-specific Lcn2 deletion could be more informative. Moreover, it is also possible that a pathogenic role for Lcn2 cannot be efficiently dissected in animal models, as has been shown for many other genes (4). In this context, a very possible role of Lcn2 in iron sequestration and microbiome regulation (18) cannot be likely examined in modeled mice, given their sterile and controlled living conditions, as well as due to the species populating the lung that are not amenable to the suggested bacteriostatic functions of Lcn2 (38, 40). However, a role for LCN2 in microbiome regulation in humans remains likely and should be pursued in future clinical studies, especially since increased airway microbiota has been associated with a more rapid disease progression and a higher risk of mortality across different patient cohorts and quantification platforms (20, 41, 42).

Moreover, microbiome differences in different animal houses could explain the contradictory results on the role of Lcn2 in inflammation in mice. For example, Lcn2 has been suggested to mediate the recruitment of neutrophils and thus to stimulate pro-inflammatory signaling; however, anti-inflammatory effects have also been suggested, including M2 polarization and T_Regs_ expansion (10). We reported here no major role for Lcn2 in LPS-induced ALI, while it was recently reported that *Lcn2*^−/−^ mice had relatively increased survival than control mice following intratracheal administration of LPS (43); the contradiction could be due to experimental design, dose, and species of administered LPS, as well as the local microbiome of the animal houses. In the same context, systemic administration of LPS in *Lcn2*^−/−^ mice was reported to result in exacerbated neuroinflammatory responses (44), although an opposite role in neuroinflammation has been also suggested promoting macrophage M1 polarization (45). However, in the lungs, LCN2 was reported to deactivate macrophages resulting in impaired immune responses following pneumococcal pneumonia (46).

As an acute phase response protein, secreted by epithelial cells upon damage, and/or infiltrating neutrophils, it is conceivable that LCN2 may contribute to chronic damage responses via the lung epithelium in IPF patients through the amplification of neutrophil recruitment. Increased neutrophils were detected in the IPF cohort examined here (Table 1; *p* = 0.038), while BAL neutrophilia has been proposed as an independent predictor of early mortality in IPF patients (47). Additionally, a high neutrophil to lymphocyte ratio (NLR) as measured from complete blood counts has also been associated with increased mortality in IPF (48). LCN2 has been shown to promote the formation of neutrophil extracellular traps (NETs) (49), which have been implicated in the pathogenesis of several diseases including IPF (50). In skin psoriasis, the amplification loop of LCN2 parallel to neutrophil-produced extracellular NETs was shown to participate in the enhancement and persistence of the local inflammatory response (51). The proinflammatory activity of NETs and LCN2 induction in psoriasis was suggested to be dependent on TLR4/IL-36R crosstalk and MyD88/nuclear factor-kappa B (NF-kB) downstream signaling (51).

Overall, although the possible role for LCN2 in IPF pathogenesis remains obscure, the acute increase in Lcn2 expression following both LPS-induced ALI and BLM-induced pulmonary inflammation and fibrosis suggests that Lcn2 is an acute phase protein of lung damage in mice, as previously suggested for acute kidney injury (9) and acute exacerbation of cystic fibrosis (52), correlating with epithelial damage and neutrophilic infiltration. Moreover, the increased LCN2 mRNA levels detected in IPF patients suggest that LCN2 levels can be used as surrogate biomarkers of pulmonary inflammation and a possible indicator of compromised pulmonary functions, urging for larger studies.

## Data availability statement

The raw data supporting the conclusions of this article will be made available by the authors, without undue reservation.

## Ethics statement

The studies involving humans were approved by Ethics Committees of the University Hospital of Heraklion (IRB numbers: 1045 and 17030). The studies were conducted in accordance with the local legislation and institutional requirements. Written informed consent for participation in this study was provided by the participants' legal guardians/next of kin. The animal study was approved by Institutional Animal Ethical Committee (IAEC) of Biomedical Sciences Research Center Alexander Fleming (#373/375), as well as the Veterinary Service and Fishery Department of the local governmental prefecture (#5508).

## Author contributions

AG performed most presented experiments and analyzed the relative data, assisted by IB, PK, KT, TK, and AT. KN and SG performed FACS. ET performed human ELISAs. DF performed *in silico* data re-analysis and supervised all statistical analyses. KA led the relative clinical protocol and provided all human samples. AG, DF, ET, and VA wrote the article, which was critically read by all co-authors. All authors contributed to the article and approved the submitted version.
