# Supplementary material for: Increased lipocalin-2 expression in pulmonary inflammation and fibrosis
Source: Front Med (Lausanne). 2023 Sep 7;10:1195501. doi: 10.3389/fmed.2023.1195501 (PMC10513431; doi:10.3389/fmed.2023.1195501)
Supplement: Supplementary file 1 [file Data_Sheet_1.PDF]

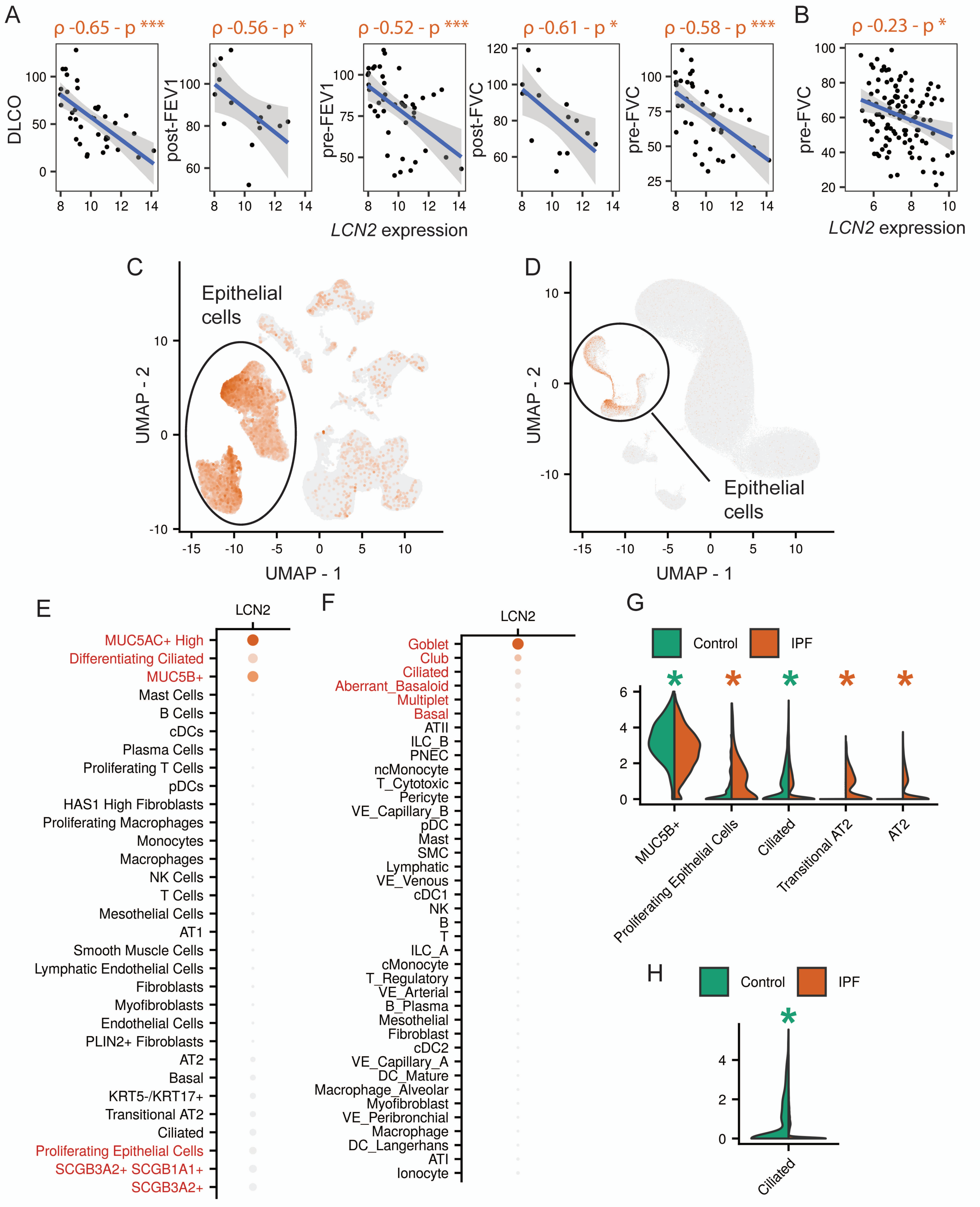

Figure S1. Increased *LCN2* expression in IPF lungs and negative relation with lung function measurements. A-B) Spearman correlation plots of *LCN2* expression with spirometry measurements in two different cohorts of patients (GSE47460_GPL6480) **(A)**, (GSE32537) **(B)**. **C-D)** UMAP plots of the single cell datasets of (Habermann, Gutierrez et al. 2020) and (Adams, Shupp et al. 2020), respectively, corroborating the epithelial origin of *LCN2* in IPF and control lungs as observed in Fig.1D. **E-F)** Dot plots of the datasets of (C-D), respectively, revealing similar findings with Fig.1E regarding the cell type specificity of *LCN2*. Red boxes highlight the cell types in which *LCN2* expression is found increased compared to the rest of the cells (marker gene) (Wilcoxon rank sum test; FC>1.2; Bonferroni adjusted p<0.05). **G-H)** Within cell types and between phenotypes differential expression proposes similar results with Fig.1F (Wilcoxon rank-sum test; * |FC|>=1.2; Bonferroni-corrected p-value<0.05; * up-regulated in IPF; * down-regulated in IPF).

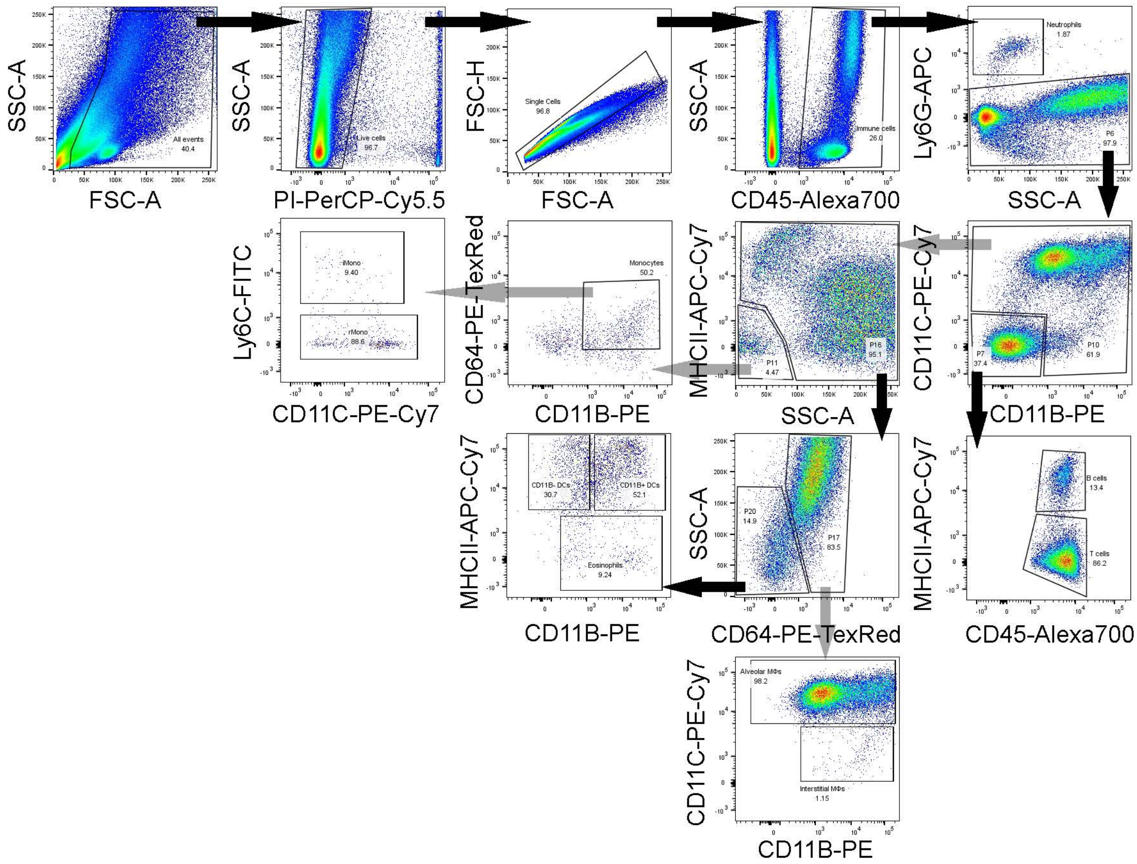

Figure S2: FACS gating strategy for murine BALF cells. The gating panel begins with the exclusion of dead cells (debris-P1), with live/dead staining (propidium iodine, PI) (P2) and doublets (P3). Immune cells were identified using the expression of CD45 (P4). The identification of neutrophils occurred using with the neutrophil marker Ly6-G. Positive staining for CD11b or CD11c on non-neutrophils is then used to distinguish the remaining myeloid leukocytes (P10) from double negative (CD11b$^-$, CD11c$^-$) lymphoid cells (P7). The lymphoid cells are then divided in MHCII (IA/IE)$^+$ B cells and MHCII (IA/IE)$^-$ T cells. The myeloid leukocytes are then subdivided based on their MHCII expression and their side scatter. The IA/IE-SCClo population (P11) contains NK cells, and monocytes. These can be distinguished based on their expression of CD11b and CD64: NK cells are CD11bint CD64-, and monocytes are CD11bhi CD64int. The monocytes are then divided again into Ly6Chi CD11c$^-$ inflammatory monocytes and Ly6C$^-$ CD11c$^+$ resident monocytes. The remaining populations in P16 (SSChi or IA/IE+) are macrophages, DCs and eosinophils. To further distinguish these populations, we used the expression of CD64 and their side scatter, CD64$^+$ macrophages can be discerned from the other populations (P17) and then be subdivided into CD11b$^-$ CD11c$^+$ alveolar macrophages and CD11b$^+$ CD11c$^-$ interstitial macrophages. Finally, P20 is then gated using CD11b vs IA/IE where CD11b$^-$ IA/IE$^+$ DCs, CD11b$^+$ IA/IE$^+$ DCs and CD11b$^+$ IA/IE- eosinophils are found.

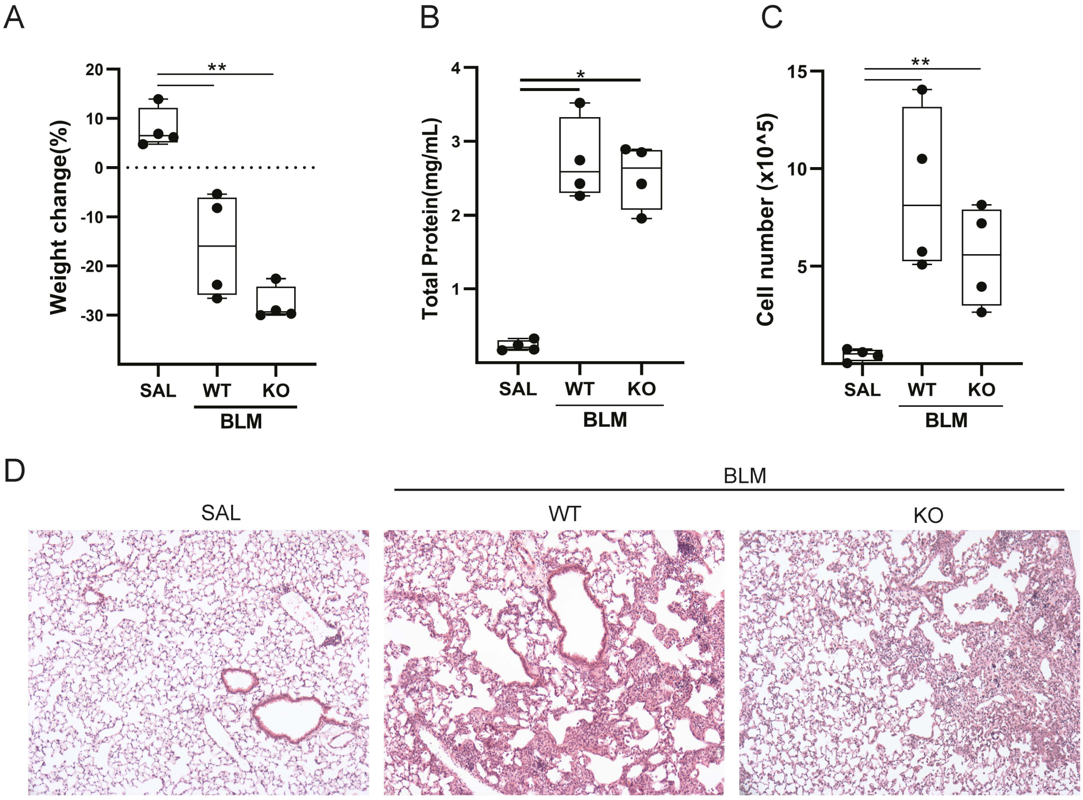

Figure S3. ***Lcn2*** **genetic deficiency has minor effects in BLM-induced pulmonary fibrosis in obese mice.** Mice were on a high fat died (HFD; 60% fat VHFD, D12492i, Research diets, New Brunswick, New Jersey, USA) for 11 weeks right after their ablactation. HFD was provided during the BLM model as well. **A)** Weight change graph from WT and KO mice post-BLM administration. **B)** Total protein concentration in BALF, as determined with the Bradford assay. **C)** Cell number in BALF of obese mice 14 days upon BLM administration, as counted with a hematocytometer; statistical significance was assessed with Kruskal-Wallis test, **denotes p<0,01. **D)** Representative H&E-stained sections of murine lungs of WT and Lcn2 KO mice (×10).
