# Supplementary material for: Increased lipocalin-2 expression in pulmonary inflammation and fibrosis
Source: Front Med (Lausanne). 2023 Sep 7;10:1195501. doi: 10.3389/fmed.2023.1195501 (PMC10513431; doi:10.3389/fmed.2023.1195501)
Supplement: Supplementary file 2 [file Data_Sheet_2.PDF]

Table S1. ***LCN2*** **RNA was detected in increased abundance in multiple** ***IPF_vs_Ctrl*** **and** ***Bleomycin_vs_Ctrl*** **lung datasets hosted in Fibromine.**

Species	PMID	Summary	Dataset	Platform	# (Exper/ Control)	*LCN2* FC	FDR
Homo sapiens	26560100	IPF lung samples were compared to healthy ones collected during an exploratory surgery.	GSE47460	GPL6480	28/15	4.50	1.17E-05
			GSE47460	GPL14550	84/75	3.86	9.48E-19
	23783374	LTRC pulmonary fibrosis samples transcriptome was compared to that of healthy controls.	GSE32537	GPL6244	115/44	2.28	2.46E-08
	19363140	Lungs of patients collected during pulmonary transplant compared with control ones derived from cancer free lung margins.	GSE10667	GPL4133	21/14	3.58	9.10E-03
	30111332	Fresh frozen lung samples from IPF patients were compared to normal lung tissue flanking pulmonary cancer areas.	GSE110147	GPL6244	22/11	2.33	1.53E-03
	21360508	Microarray data from IPF patients were juxtaposed to data from control individuals.	GSE48149	GPL16221	12/5	6.15	2.32E-02
	25217476	Differences between IPF and control transcriptomic profile were examined based on lung biopsies or explants.	GSE53845	GPL6480	39/7	5.43	3.97E-04
	28230051	LTRC IPF and control samples were examined to identify transcriptional differences between disease and steady state conditions.	GSE92592	GPL11154	19/18	6.77	5.69E-11
	16998095	Sporadic idiopathic interstitial pneumonia samples were compared to control samples.	GSE5774	GPL4255	13/17	-1.85	1.3E-01
	28081703	RNA-seq assessed the transcriptome of IPF and control FFPE lung samples.	GSE83717	GPL11154	6/5	2.83	1.4E-01
	29329637	IPF samples were compared with healthy controls using RNA-seq.	GSE99621	GPL16791	8/8	1.75	1.7E-01
	21974901	Healthy samples obtained during lung transplantation were compared to IPF.	GSE24206	GPL570	8/6	3.36	2.0E-01
	24647608	mRNA of IPF and control individuals was examined using next generation sequencing.	GSE52463	GPL11154	6/4	2.25	2.3E-01
	21241464	LRTC IPF and uninvolved lung samples of cancer and a transplant patient were compared.	GSE21369	GPL570	11/6	1.92	4.3E-01
	26453058	Control samples from patients with primary spontaneous pneumothorax was compared to samples originating from IPF patients.	GSE72073	GPL17586	4/3	1.44	4.6E-01
Mus musculus	23648810	Total RNA was collected from C57BL/6 mouse lungs treated or not with bleomycin. (BlmD14_vs_Ctrl)	GSE37635	GPL6885	4/6	5.35	7.82E-06
	21602491	Mice treated for 21 days with bleomycin were compared to saline treated control ones. (BlmD21_vs_Ctrl)	GSE25640	GPL1261	3/3	3.03	3.25E-02
	19966781	Affymetrix microarrays were used to assess murine transcriptome 14 days post to bleomycin instillation compared to control untreated samples. (BlmD14_vs_Ctrl)	GSE18800	GPL1261	3/4	4.22	2.67E-04
	23459460	Bleomycin-treated mice were compared to PBS-treated ones to assess differences in their transcriptomic profiles. (BlmD14_vs_Ctrl)	GSE34814	GPL13912	3/3	3.01	2.32E-03
	23565148	Male C57BL6/J mice received bleomycin or saline treatment. (BlmD14_vs_Ctrl)	GSE40151	GPL1261	16/16	-1.66	1.89E-02
	19652365	Mice receiving bleomycin via oropharyngeal instillation were compared to control counterparts. (BlmD14_vs_Ctrl)	GSE16846	GPL339	3/3	4.29	3.42E-02
	-	Mice received bleomycin or water via tracheal injection. (BlmD21_vs_Ctrl)	GSE77326	GPL15887	6/6	1.41	3.47E-02

Platform: *Unique gene expression inquiring technology IDs;* ***FC****: Fold Change;* ***LTRC****: Lung Tissue Research Consortium;* ***Red font*** *marks datasets of Fig.1B & Fig.2B;* ***Dotted line*** *separates significant (top) from insignificant (bottom) FDR values*

Table S2. Single cell datasets exploited for the assessment of *LCN2/Lcn2* cell specificity.

PMID	Summary	Dsataset	Comparison	# Exp / Control	# Cells	# Genes	Figure
33650774	Transcriptome examination of IPF or control origin single cells based on the integration of three independent cohorts.	github	IPF_vs_Ctrl	19/29	181814	49498	Fig1
32832599	Transcriptome interrogation of whole lung dissociates at the single cell level.	GSE136831	IPF_vs_Ctrl	32/28	243472	45947	FigS1
32832598	Single cell exploration of non-fibrotic and pulmonary fibrosis lung samples.	github	IPF_vs_Ctrl	12/10	89326	33694	FigS1
32678092	Time-course experiment of bleomycin-treated and control mice at a single cell resolution.	github	Blm_vs_Ctrl	21/7	29142	23400	Fig3

Blm: *Bleomycin*

Table S3. ***LCN2*** **and** ***Lcn2*** **expression mark neutrophil and epithelial cells of several organs in both human and mouse species as revealed by CellMarker2.0 data mining.**

Species	PMID	Tissue	Cell type	Evidence
Homo sapiens	34128959	Blood	Neutrophil	Experiment
	31753849	Colon	Progenitor cell	Experiment
	31604275	Kidney	Pelvic epithelial cell	Experiment
	33730555	Lacrimal gland	Ductal cell	Experiment
	33619773	Liver	Cholangiocyte	Experiment
	34721400	Peripheral blood	Polymorphonuclear myeloid-derived suppressor(PMN-MDSC) cell	Experiment
	35111153	Peripheral blood	Neutrophil progenitor cell	Experiment
Mus musculus	32424270	Blood	Granulocytic neutrophil	Experiment
	33028096	Blood vessel	Fibroblast	Experiment
	35175542	Blood vessel	Neutrophil	Experiment
	29466336	Bone marrow	Neutrophil	Single-cell sequencing
	29915358	Bone marrow	Stage I neutrophil	Single-cell sequencing
	32221280	Brain	Choroid cell	Experiment
	32640942	Brain	Neutrophil	Experiment
	33028409	Brain	Microglial cell	Experiment
	32759216	Fetal ovary	Blood-related cell	Experiment
	29622724	Kidney	Neutrophil	Single-cell sequencing
	34367152	Kidney	Neutrophil	Experiment
	24739965	Lung	Type II pneumocyte	Single-cell sequencing
	34389631	Lung	Neutrophil	Experiment
	34301765	Meninge	Neutrophil	Experiment
	32059779	Testis	Endothelial cell	Experiment
